# The London Panel Committee: An Important Series of Decisions

**Published:** 1914-12-19

**Authors:** 


### THE LONDON PANEL COMMITTEE.

### An Important Series of Decisions.

Many important matters occupied the attention of the London Panel Committee at its meeting on December 15.

Representation of Non-Panel Interests.

In the first place, efforts were renewed towards giving definite shape to the opinion expressed both by the local Medical and Panel Committees, that the membership of the two bodies should be identical. The Panel Committee agreed to invite a conference with the local Medical Committee, and as its contribution towards a friendly arrangement proposed to vary its rule dealing with co-option of outside practitioners to provide that there should be added to the Panel Committee twelve members, chosen as follows:

(i.) Three representatives of the medical staffs of hospitals. (ii.) Two representatives of medical officers of health, one chosen from the north, and one from the south side of the Thames.

(iii.) Three representatives of non-panel practitioners north of the Thames, and three from the south of the Thames. (iv.) One representative of women practitioners on the panel.

It was urged that such an arrangement would make for unity in the profession, and that, as sixty-two out of the seventy-seven members of the Committee would be elected, under the new arrangement, by the panel practitioners, no serious objection would be likely when the proposal was submitted to the Panel Committee electorate.

Relations with the B.M.A.

Discussion also took place with reference to overtures by the British Medical Association, which, in a circular-letter, asked the Panel Committee to consult the Association in its difficulties, and enter into close and steady relationship with the central office. The Sub-Committee advised the Committee to accede fully to this request.

Dr. Cowie, however, moved an amendment that the friendly relations with the Association be restricted to an exchange of documents, and only be extended gradually as the Panel Committee found that its independence was not being undermined. Dr. Major Greenwood, Dr. Lauriston Shaw, Dr. Batten, and others urged that the Association should be met quite fully and supported as the only body able to fight the battle of the medical profession.

The amendment was lost, whereupon Dr. J. A. Angus moved a further amendment that panel practitioners who were not members of the Association be given representation on the Committee of the Association dealing with Insurance Act matters, and that this be a condition antecedent to the support of the Panel Committee. In reply, it was urged that the Association could not legally place on its Committees those who were not members, and Dr. Denning twitted those practitioners who had left it to their brethren in the Association to bear the cost of the Insurance Act campaign. Only three voted for the amendment, and the draft scheme, as above, was referred to the proposed conference.

Principles of Tuberculosis Schemes.

The Committee adopted a series of resolutions calling upon the Local Government Board to secure that a scheme for dispensary treatment of tuberculosis is prepared immediately in every borough where a scheme does not already exist, and expressing the opinion that the consulting officer should be an expert in tuberculosis and experienced in clinical work, and that a medical officer of health should not in any circumstances act in that capacity; also that where a dispensary scheme did not exist, the medical adviser to the Insurance Committee should act as "consulting officer" for domiciliary treatment.

Ethics of the Panel List.

The Insurance Committee asked if the Panel Committee would concur in a proposal that a practitioner should not be permitted to have his name included in a borough panel for an area beyond two miles' radius from his residence. In a discussion it was suggested that names were sometimes put on several borough panels for advertisement purposes. On the other hand it was urged that doctors frequently visited patients who had removed to distant boroughs and put their name on the local list in order to treat insured persons in the same households; also that when living on the borders of three boroughs it was necessary for a doctor to be on the list for each. Dr. Major Greenwood moved that the suggestion of the Insurance Committee be accepted on the ground that it would show practitioners to be serious in their objection to being liable to visit beyond a two-mile radius. Dr. Lauriston Shaw objected to allowing the Insurance Committee to act for the Panel Committee, which should enforce its own disciplinary measures if the present practice were abused. Ultimately it was decided to inform the Insurance Committee that no alteration should be made in the present practice.

